# Role of salivary leukocyte protease inhibitor in periodontal disease progression

**DOI:** 10.4103/0972-124X.70830

**Published:** 2010

**Authors:** Deepak Pateel, H. Seema, Akla Kale

**Affiliations:** *Department of Oral Pathology, K. M. Shah Dental College and Hospital, Sumandeep Vidhyapeeth University, Vadodara, Gujarat, India*; 1 *KLE’s Vishwanath Katti Institute of Dental Sciences, KLE University, Belgaum, India*

**Keywords:** Chronic periodontitis, protease, saliva, SLPI

## Abstract

**Context:**

Proteases play a major role in the tissue destruction involved in periodontal disease. It is known that the balance between proteases and their inhibitors is a major determinant in maintaining tissue integrity. The association between the proteases and periodontitis is well established, but not many studies have been carried out to know the role played by a protease inhibitor like salivary leukocyte protease inhibitor (SLPI) in periodontitis.

**Aim:**

The aim of the present study was to correlate SLPI with periodontitis.

**Settings and Design:**

Case-control study.

**Materials and Methods:**

Seventy-five clinically confirmed cases of periodontitis and 20 controls were included in the study. A detailed case history and periodontal index (PI) were recorded. Two milliliters of unstimulated saliva samples was obtained and subjected to quantification of SLPI leaves using SLPI in enzyme-linked immunosorbent assay (ELISA) kit. Based on the periodontal index score of the individuals, the cases and controls were divided into groups A, B and C, and the obtained SLPI levels were compared among the groups.

**Statistical Analysis:**

Mann-Whitney *U* test and correlation coefficient test.

**Results:**

The results showed that in the initial stages of periodontitis there is a tendency of SLPI levels to be raised. The SLPI levels were found to be reduced in the terminal stages of periodontitis.

**Conclusion:**

It appears that SLPI accumulates in the local environment, at least in the initial stages of the periodontal disease, probably to inhibit the action of increased elastic activity.

## INTRODUCTION

Oral cavity is a unique environment. Oral mucosa is a critical protective interface between external and internal environments and serves as a barrier to the myriad microbiological species present in this warm, moist environment. The periodontium surrounding the tooth is specialized to form an attachment and seal around each tooth. This unique function imparts special challenges to the periodontium and makes it vulnerable to periodontal diseases, especially in view of continuous exposure to bacterial biofilm.[1]

Periodontitis is the most prevalent oral disease, affecting almost 90% of the population, and is also the most frequent cause of tooth loss in India.[2] Elastase plays a significant role in the connective tissue destruction associated with inflammatory processes. The free elastase activity, as well as that of other proteases, is regulated by specific inhibitors that are either produced locally or circulate in the plasma.[3] Such inhibitors include SLPI, α1 protease, α1 antitrypsin (α1-AT), α2 microglobulin, etc.[4] SLPI, a major leukocyte protease inhibitor, is an acid-stable, highly basic, nonglycosylated protein associated with a number of glandular secretions, including saliva. It is present at an average concentration of 1 to 10 μg/mL in saliva. The main functions of SLPI are as follows.[5]

1. Protects the local tissues against determinant consequences of inflammation. It inhibits the action of proteases such as cathepsin G, elastases, trypsin, PMNs chymotrypsin and trypsin from acinar cells and chymase from mast cells.
2. Shields the tissues against inflammatory products by down-regulating the macrophage response against bacterial lipopolysaccharide [LPS].
3. Decreases the C5a-related chemotactic activity.
4. SLPI has inhibitory activity against bacteria, fungi and viruses.
5. Promotes cutaneous wound healing.

The diagnosis of active phase of periodontal disease and identification of patients at risk of active disease are challenges for clinical investigators and clinicians. Clinical indicators may reflect only past inflammation and cannot distinguish between active and inactive sites.[3] Saliva plays a protective role in maintaining oral health and consists of many locally and systemically derived factors that can be used as the basis for diagnosis of periodontal disease status. The proposed markers for diagnosing the disease status include proteins of host origin (enzymes, immunoglobulins, SLPI), phenotype markers (keratins), host cells, hormones, bacteria and bacterial products.[3]

There is fast-growing evidence that the body’s own immune system is challenged by exacerbating damage caused by periodontal disease. Thus the accumulation of SLPI in the local environment may represent an intrinsic feedback mechanism to prevent harmful effects of inflammation.[7] It is generally postulated that the balance between the proteinase and antiproteinase is a prerequisite for the maintenance of tissue integrity, and any disturbance in this equilibrium will cause active destruction of tissues. Reports have shown that the SLPI concentration is altered in various inflammatory diseases.[8] Thus the present study was carried out to compare compare between different stages of periodontitis and correlate the SLPI levels with the status or severity of periodontitis and to determine the role played by SLPI in periodontal disease progression.

## MATERIALS AND METHODS

### Source of data

The present study was carried out at our institution. The study group comprised of 75 subjects with chronic periodontitis, and control group comprised of 20 subjects with normal periodontium.

### Inclusion criteria

Patients with periodontitis were chosen for the study based on the following criteria.

1. Subjects with minimum of 20 teeth
2. Subjects with minimum of 4 periodontal pockets
3. Subjects with age ranging from 25 to 65 years

### Exclusion criteria

Subjects less than 25 years of age.

Subjects with a history of consumption of antibiotics for the last 2 months.

Subjects with known salivary gland disease and other inflammatory lesions.

### Method of collection of data

After obtaining an informed consent, a detailed case history and Russell’s periodontal index (PI) were documented. Based on the PI score of the individuals, the patients and controls were divided into the following groups [Table 1].

**Table 1:** Groups according to PI scores of patients and controls

PI score	Interpretation
PI score of 0-1.9	Group C [control group]
PI score of 2-4.9	Group A [established periodontal disease]
PI score of 5-8	Group B [terminal periodontal disease]

### Collection of saliva samples

Patients were asked to rinse their mouth thoroughly with water. Unstimulated whole saliva was collected in a sterile bottle by asking the patient to expectorate into it gradually over a period of 5 to 10 minutes. Approximately 1-2 mL of saliva was collected.

### Estimation of SLPI levels

The obtained saliva samples were stored in deepfreeze at –20°C until further test was carried out. The SLPI levels were estimated by using SLPI ELISA test kit (Hycult Biotechnology). The human SLPI ELISA test kit is a solid-phase enzyme-linked immunosorbent assay based on the sandwich principle. Samples and standards are incubated in microtiter wells coated with antibodies recognizing human SLPI. The human SLPI was captured by solid bound antibody. Unbound material present in the sample was removed by washing. Next, biotinylated second antibody tracer to human SLPI was added to the wells. In the presence of SLPI in sample, the tracer antibodies bind to the captured SLPI. The excess tracer was removed by washing. Next a streptividin-peroxidase conjugate was applied to the wells. The conjugate reacts specifically with the biotinylated tracer antibody bound onto the detected SLPI. The excess streptividin-peroxidase conjugate was removed by washing, and substrate tetramethylbenzidone (TMB) was added to the wells. Color develops proportional to the amount of human SLPI present in the sample. The enzyme reaction was stopped by the addition of citric acid, and the absorptions at 450 mm were measured with spectrophotometer.

### Statistical analysis

The obtained periodontal index scores and SLPI levels in both study and control groups were tabulated, and statistical analysis was performed using Mann-Whitney *U* test and correlation coefficient test. Differences in mean SLPI levels between control and study groups were evaluated based on periodontal index scores of the groups with the Mann-Whitney *U* test and correlation coefficient test. In all the above tests, a *P* value of less than .05 was accepted as indicating statistical significance.

## RESULTS

Maximum number of patients affected by periodontitis were in the fourth, fifth and sixth decade of their life, that is, 25.33% (19 patients) in the age group 31-40 years, 34.66% (26 patients) in age group 41-50 and 29.33% (22 patients) in age group 51-60 years. Seventy percent (14) of the controls were in the age group 41-50 years; and 30% (6), in the age group 31-40 years [Table 2].

Of the 75 patients included in the study, 33.33% (40) were males and 46.66% (35) were females. In the control group, out of 20 patients, 65% (13) were males and 35% (7) were females [Table 3].

**Table 2:** Age distribution of patients and controls

Age range	No. of patients in study group	Percentage	No. of patients in control group	Percentage
21 - 30	04	5.33		
31 - 40	19	25.33	6	30
41 - 50	26	34.66	14	70
51 - 60	22	29.33		
61 - 70	04	5.33		

Maximum number of patients affected by periodontitis were in the fourth, fifth and sixth decade of their life, that is, 25.33% (19) in age group 31-40 years, 34.66% (26) in age group 41-50 and 29.33% (22) in age group 51-60. Seventy percent (14) of the controls were in the age group 41-50 years; and 30% (6), in the age group 31-40 years

**Table 3:** Gender distribution of patients and controls

Sex	No. of cases	Percentage	No. of controls	Percentage
Male	40	53.33	13	65
Female	35	46.66	7	35

Of the 75 patients included in the study, 33.33% (40) were males and 46.66% (35) were females. In the control group, out of 20 patients, 65% (13) were males and 35% (7) were females.

The periodontal index scores of the groups are shown in Tables 4–6. A statistically significant difference with *P*=.086 was found in the SLPI levels between group B (terminal periodontal disease) and group C (controls) subjects. The difference in the SLPI levels between group A and group B and between group A and group C was not found to be statistically significant [Table 7].

**Table 4:** Periodontal index scores and SLPI levels in study group A

Periodontal index scores	Salivary SLPI levels (μg/mL)
3.3	10.932
3.56	11.941
4	10.258
3.8	2.328
2.7	10.791
3.18	5.918
3.93	7.294
2.689	7.172
2.26	3.588
2.6	3.119
4.4	15.911
3.6	3.498
3.2	2.701
4.5	1.358
2.56	3.289
3.3	2.666
3.3	2.58
4.1	8.9
3.68	3.505
2.4	2.504
3.5	12.298
3	3.198
4	3.391
2.8	2.709
2.6	4.936
2.9	1.713
2.6	10.862
3.14	0.9679
3	3.948
3.2	3.244
2.9	4.998
3.1	2.997
3.5	0.713
3.2	8.5
2.6	7.791
2.7	0.492
3.9	3.904
3.2	6.785
3	4.436
3.1	7.598
4.3	13.735
3.5	8.776
4	2.252
2.7	3.137
3.2	9.868
4	3.162
3.75	10.941
2.4	2.697
2.5	7.603
2.57	5.244

**Table 5:** Periodontal index scores and SLPI levels in study group B

Periodontal index scores	Salivary SLPI levels (μg/mL)
5.36	4.053
6.4	2.712
5.87	11.954
7.6	3.281
6.6	2.301
5.1	2.879
5	9.226
5.6	4.286
6.7	8.713
5.6	1.176
6.5	6.789
7.3	1.916
6.24	2.532
5.5	1.022
6.5	4.416
5	10.834
5.7	3.214
6.5	3.143
6.9	2.536
5.6	2.394
5.9	0.668
6	3.399
5.5	0.775
6.1	2.985
6.37	13.687

**Table 6:** Periodontal index scores and SLPI levels in control group

Periodontal index scores	Salivary SLPI levels (μg/mL)
1.81	4.522
1.875	3.995
1.851	7.511
1.78	8.228
1.81	5.233
1.875	4.688
1.78	3.33
1.78	4.118
1.875	3.245
1.78	4.41
1.75	6.616
1.7142	4.326
1.71	6.465
1.625	3.807
1.571	5.605
1.75	6.571
1.75	3.865
1.714	5.667
1.625	4.331
1.571	5.503

**Table 7:** SLPI levels in different groups

	Group A	Group B	Group C
Mean SLPI	5.6630	4.4356	5.2518
Mean PI scores	3.3284	6.576	1.7498

P for SLPI levels compared between group A and group C=.3981 (NS-Not Significant); P for SLPI levels compared between group B and group C=.0086 (VS, very significant); P for SLPI levels compared between group A and group B=.1080 (NS); A statistically significant difference, with P=.086, was found in the SLPI levels between group B (terminal periodontal disease) and group C (controls); The difference in the SLPI levels between group A and group B and between group A and group C was not found to be statistically significant

## DISCUSSION

In the present study, the maximum number of patients, i.e., 34.6% (26), was in the 41-50 years age group. In the control group, 70% (14) were in the 40-50 years age group [Table 2]. This is in accordance to various cross-sectional surveys, such as National Health and Nutritional Examination Surveys (NHANES 1988-1994), according to which the prevalence of chronic periodontitis and severity of attachment loss increase with age, from a low of 35.7% for 30-39 years age group to a high of 80% for 60-69 years age group.[2,9]

In the present study, in the study group, the SLPI concentration ranged from 0 to 12 μg/mL, whereas in the control group the SLPI level ranged from 3 to 9 μg/mL [Table 4,5 and 6]. Very few studies are available on the mean SLPI levels in normal subjects and in patients of periodontitis. The mean SLPI concentration reported in various studies ranged from 4 to 24 μg/mL, 1.17 μg/mL, 0.7 μg/mL and 0.2 μg/mL.[10–14] But the physiologic range of SLPI concentration reported in the literature is 1-10 μg/mL.[5] This variation could be due to variation in the methodology applied for the estimation of SLPI concentration in different studies.

The mean concentration of SLPI in control group was found to be 5.235 μg/mL. Among the study groups, in group A [established periodontal lesion group] the mean SLPI concentration was found to be 5.633 μg/mL; and in group B [terminal periodontal disease group], it was 4.425 μg/mL.

The mean SLPI concentration in group A was found to be increased when compared to the control group. The difference in the concentration of SLPI between group A and control group was statistically insignificant (*P*= .382).

Studies have established that SLPI levels are significantly increased during gingivitis and periodontitis.[10,11] Experiments by Gillian *et al*. have shown that SLPI plays a critical role in the control of excessive tissue destruction and mediates wound healing.[15] Experiments have also demonstrated that the neutrophil elastase is involved in the initial destruction of periodontal ligament during early stages of periodontal disease.[16] Reports have shown that the SLPI plays an important role in controlling salivary elastase activity.[17] These findings support the findings from the present study, viz., increased SLPI levels in group A and control group when compared to group B

The mean SLPI concentration in group B (4.425 μg/mL) [terminal periodontal disease] was significantly reduced when compared to the control group [5.325 μg/mL], with a *P* value of .008. Studies have reported that cathepsins and bacterial cysteine proteases are involved in the degradation of SLPI, and a proportion of SLPI would also get consumed in the resulting complex with elastase.[18,11] So it appears that SLPI levels are decreased in the late stages of periodontitis.

The mean SLPI concentration in group A [established periodontal disease] was increased when compared to group B [terminal periodontal disease], with a *P* value of .1080, which was statistically not significant. Various studies have demonstrated the importance of SLPI in regulating the activity of serine proteases that are released during inflammation. Recently it has been shown that these inhibitors also play a role in tissue repair and extracellular matrix synthesis.[19] The findings, along with the reports of increased elastase activity during periodontitis,[20] indicate that our body responds to the situation by secreting more and more SLPI into the local environment during the active stages of periodontal disease.

From the results obtained in the present study, it appears that SLPI plays a role in gingivitis and periodontitis, at least in the early stages of these conditionsit appears that SLPI plays a role in gingivitis and periodontitis, atleast in the early stages of these conditions, where active destruction of tissue is taking place. Because of its suggested antiproteolytic, antimicrobial and anti-inflammatory profiles, SLPI probably plays a protective role by maintaining a balance between proteases and antiproteases.

We have also found that SLPI levels are reduced considerably in the terminal stages of periodontitis. It will be interesting to see if analysis of SLPI levels can aid in screening of patients with terminal periodontal disease.

Finally, as host modulation as a treatment strategy in the treatment of periodontal disease is gaining importance in recent times, it appears that SLPI could possibly have a therapeutic role as it facilitates necessitates the up-regulation, inducement or enhancement of repair and wound healing in conditions such as periodontitis.

## CONCLUSION

From the results of our study, it is evident that in the initial stages of periodontitis there is a tendency of SLPI levels to be raised. It appears that SLPI accumulates in the local environment, probably to inhibit the action of increased elastic activity. It could also be due to other protective functions performed by the SLPI, like antimicrobial, anti-inflammatory, tissue repair and wound healing, during periodontal disease progression.

SLPI levels were found to be reduced in the terminal stages of periodontitis. This could be due to the degradation of SLPI by cathepsin L and bacterial cysteine protease and formation of complex with elastase.

### Recommendations

Further studies are necessary to further substantiate the findings of the present study. Studies to investigate the role played by elastase, SLPI and cathepsins in both saliva and gingival cervicular fluid of periodontitis subjects are required. Large multi-center trials are needed to evaluate the role of SLPI as a host-modulating agent in the treatment of periodontitis.
